# Comparative Physiological and Transcriptional Analyses of Two Contrasting Drought Tolerant Alfalfa Varieties

**DOI:** 10.3389/fpls.2015.01256

**Published:** 2016-01-12

**Authors:** Wenli Quan, Xun Liu, Haiqing Wang, Zhulong Chan

**Affiliations:** ^1^Key Laboratory of Plant Germplasm Enhancement and Specialty Agriculture, Wuhan Botanical Garden/Sino-Africa Joint Research Center – Chinese Academy of SciencesWuhan, China; ^2^University of Chinese Academy of SciencesBeijing, China; ^3^Key Laboratory of Adaptation and Evolution of Plateau Biota, Northwest Institute of Plateau Biology – Chinese Academy of SciencesXining, China

**Keywords:** alfalfa, drought stress, stomata density, lateral root, physiological changes, transcriptional expression

## Abstract

Drought is one of major environmental determinants of plant growth and productivity. Alfalfa (*Medicago sativa*) is a legume perennial forage crop native to the arid and semi-arid environment, which is an ideal candidate to study the biochemical and molecular mechanisms conferring drought resistance in plants. In this study, drought stress responses of two alfalfa varieties, Longdong and Algonquin, were comparatively assayed at the physiological, morphological, and transcriptional levels. Under control condition, the drought-tolerant Longdong with smaller leaf size and lower stomata density showed less water loss than the drought-sensitive Algonquin. After exposing to drought stress, Longdong showed less severe cell membrane damage, more proline, and ascorbate (ASC) contents and less accumulation of H_2_O_2_ than Algonquin. Moreover, significantly higher antioxidant enzymes activities after drought treatment were found in Longdong when compared with Algonquin. In addition, transcriptional expression analysis showed that Longdong exhibited significantly higher transcripts of drought-responsive genes in leaf and root under drought stress condition. Taken together, these results indicated that Longdong variety was more drought-tolerant than Algonquin variety as evidenced by less leaf firing, more lateral root number, higher relative aboveground/underground biomass per plant and survival rate.

## Introduction

Drought stress is a major environmental factor limiting plant growth, development, and survival rate, leading to enormous yield loss. It is estimated that the arid and semi-arid regions account for approximately 30% of the total worldwide area (Sivakumar et al., 2005). Water deficiency has become a severe threat to sustainable agriculture (Castroluna et al., 2014). Accordingly, higher plants have evolved complex mechanisms to rapidly adapt to drought stress conditions. In recent years, stress physiology in crops has become one of the central issues of plant biology and more attentions have been paid to mechanisms of plant stress tolerance, including physiological changes biochemical metabolisms, morphological variations, and gene expression regulation. These studies further help us develop different genetic methods to improve plant stress tolerance and prevent crop yield loss (Shi et al., 2012a; Castroluna et al., 2014).

Alfalfa (*Medicago sativa*) is a perennial forage legume species with great agronomical interest, including low production cost, and high quality. Moreover, the deep root system of alfalfa helps prevent from soil and water loss in semi-dry lands (Moran et al., 1994). Therefore, alfalfa is a fairly hardy species and has a relatively higher level of drought tolerance compared with many other food crops (Tang et al., 2013). Previous agronomical, physiological, and biochemical studies strongly suggest that alfalfa is more drought tolerant than pea (Moran et al., 1994) and ureide-producing grain legumes (Sinclair and Serraj, 1995). Nonetheless, the deleterious effects of abiotic stress (such as drought and salt stresses) still represent major limit to alfalfa production (Wang et al., 2015). Recently, while long-term, traditional breeding programs to enhance alfalfa stress tolerance and improve crop yield under periodic drought are under way (Vasconcelos et al., 2008; Li et al., 2010), many transgenic alfalfa plants have been obtained for enhancing tolerance to drought stress (Zhang et al., 2012; Tang et al., 2013; Li et al., 2014; Ferradini et al., 2015). For example, co-expression of tonoplast NHX and H^+^-PPase genes from the xerophyte *Zygophyllum xanthoxylum* significantly improved the growth performance of transgenic alfalfa with more Na^+^, K^+^, and Ca^2+^ accumulation, and enhanced drought stress tolerance (Bao et al., 2015). Wang et al. (2014) suggested that transgenic alfalfa plants expressing *AtNDPK2* exhibited enhanced tolerance to high temperature, salt and drought stresses and showed better plant growth partially through increased expression of auxin-related indole acetic acid (IAA) genes under normal growth condition compared to non-transgenic plants. Overexpression of *GsZFP1* induced higher expression of stress-responsive marker genes, including *MtCOR47*, *MtRAB18*, *MtP5CS*, *and MtRD2*, in transgenic alfalfa than those of WT under drought stress condition and enhanced the drought tolerance of alfalfa (Tang et al., 2013).

As a perennial forage crop, alfalfa has a wide-ranging distribution and thus is expected to show differing levels of drought tolerance. Erice et al. (2010) showed that morphological components such as leaf area ratio (LAR), specific leaf area (SLA), and leaf weight ratio (LWR) were different in four alfalfa varieties differing in drought sensitivity and growing climate; and variety Tafilalet adapted to a Mediterranean climate exhibited higher drought resistance by decreasing LWR and SLA under progressive drought. Systems analysis of two alfalfa varieties, Wisfal and Chilean, with contrasting tolerance to drought revealed common and divergent responses to drought stress. At a qualitative level, they exhibited similar strategies to cope with drought; however, quantitative differences were observed which may contribute to greater drought tolerance in Wisfal with lower stomatal density and stomatal conductance, greater root growth, and larger accumulation of several osmolytes and antioxidants (Kang et al., 2011). The ability for growth under various conditions indicates that alfalfa is capable to develop different mechanisms of resistance to a large range of constrains, and especially to drought (Erice et al., 2010). Previous research on alfalfa focused primarily on yield, forage quality, disease resistance, winter hardiness, nodulation, photosynthesis, and metabolite, especially on transgenic alfalfa construction in recent years (Kang et al., 2011; Zhang et al., 2012; Gebril et al., 2015; Wang et al., 2015). However, comprehensive and comparative analyses of physiology, morphology and gene expression changes in alfalfa cultivars differing in drought tolerance under long-term drought stress are sparse.

This study is to better understand the capability of contrasting alfalfa varieties to long-term drought stress and to identify physiological, morphological, and transcriptional alterations for development of novel alfalfa varieties with enhanced drought resistance. For this purpose, two alfalfa varieties with different drought tolerance were used in the study to evaluate the difference of stress responses under water deficit condition by assaying plant growth, levels of reactive oxygen species, antioxidant enzyme activities, and drought-related gene expression.

## Materials and Methods

### Plant Material and Culture Conditions

Seeds of two alfalfa (*Medicago sativa* L.) varieties, Longdong, and Algonquin were kindly provided by Northwest Plateau Institute of Biology, The Chinese Academy of Sciences.

The seeds were surface-sterilized with 5% sodium hypochloride solution for 10 min, thoroughly rinsed five times with sterile Milli-Q water and stratified at 4°C for 2 days in the dark. Then, the seeds were germinated on half-strength Murashige and Skoog (MS) medium (pH 5.7) under a 16/8 h light/dark cycle, with a light intensity of 350 μmol⋅m^-2^⋅s^-1^ and a relative humidity of 65% at 25°C.

### Drought Stress Treatment

After 6 days of germination, the seedlings with the same size were selected and transferred to plastic pots (10 cm diameter at top, 7.5 cm diameter at bottom, and 8.5 cm height) filled with equal quantity pre-autoclaved vermiculite while each pot was planted nine seedlings. The plants were irrigated with similar quantities of 0.2% (w/v) nutrient solution (18–18–18 of nitrogen–phosphorus–potassium per 100 g fertilizer, plus 3% magnesium and microelement) from trays placed underneath the pots twice every week for 3 weeks. The healthy 4-week-old alfalfa plants with similar size were selected for drought tolerance test. Half of the plants were put under drought-stress imposition by withholding water in the soil for 18 days, while another half was subjected to well watering condition (control). Twelve pots of seedlings from each variety were served as replicates in each independent experiment, and all these pots of plants were conducted in a randomized complete block design under the same growth condition. In addition, the position of these pots was changed daily in order to minimize the environment effects. At 6, 12, and 18 days of drought treatment, the leaf samples were harvested and used for physiological and morphological measurement. In the end of the experiment, the drought-stressed plants were re-watered for 7 days and survival rate were calculated.

Additionally, for better observation of root development, the well-watered alfalfa seedlings with 2-week-old were treated with drought stress by withholding water. After 6 days of drought treatment, lateral root numbers of two alfalfa varieties were counted and the main root length was measured. The density of lateral root was calculated through dividing lateral root number by the main root length under control and drought conditions.

### Measurement of Water Loss and Leaf Water Content

To access leaf water status, leaf water loss *in vitro* and leaf water content (LWC) *in vivo* were measured in the study. Leaf water loss was expressed as % change in detached leaf fresh weight, and LWC was the assessment of leaf water potential *in vivo* (Shi et al., 2012a).

To analyze the water loss rate between the two alfalfa varieties, the detached leaves grown under control conditions at 12 days after drought treatment were collected and put onto the clean filter paper in the same growth room. The leaf fresh weight of the detached leaves were quantified every 1 h intervals for up to 8 h and the water loss rate was calculated from the decrease in the rate of FW at designated time intervals (Shi et al., 2012b)

For LWC analysis, the leaf samples at the same part of the plants were harvested at 6, 12, and 18 days under control and drought stress conditions, respectively. LWC was measured according to the following equation: LWC = (FW - DW)/FW × 100, where FW is the leaf fresh weight and DW the dry weight (Luo et al., 2011).

### Assay of Electrolyte Leakage

Electrolyte leakage (EL) was analyzed from the detached leaves of control and drought-stressed leaves at different intervals. The detached leaves were placed in 50 ml tubes containing 15 ml ddH_2_O and gently shanked for 6 h at room temperature. Then, the leaves were boiled at 100°C for 20 min. When the leaves were cooled to room temperature, the percentage of EL was measured by the formula EL (%) = (*C*_i_/*C*_max_) × 100, where *C*_i_ and *C*_max_, respectively, represents the conductivity before and after boiling of the detached leaves.

### Measurement of Stomatal Density

The third youngest healthy leaves from 4-week-old plants under well-watered condition were harvested and used for stomatal density measurement. The upper and lower epidermis of terminal leaflet from the same part was peeled off, observed and photographed under a microscope (MF52, Mshot Co., Guangzhou, China). Leaf margins and the areas near to the midrib were avoided. The stomata numbers were counted and the density was calculated from photomicrographs. Ten leaves from two alfalfa varieties were used for each replicate.

### Quantification of Proline Content

Proline contents of control and drought-stressed treatment were quantified as previously described (Shi et al., 2012a). Briefly, we extracted proline samples with 3% (w/v) sulfosalicylic acid, and the extractions were injected to the compounds of ninhydrin reagent and glacial acetic acid. Then, the mixture was boiled at 100°C for 40 min. When it cooled to the room temperature, the proline content was assayed through the absorbance of 520 nm.

### Determination of Ascorbate, H_2_O_2_, and Antioxidant Enzyme Activities

The fresh leaf samples were ground quickly with liquid nitrogen and homogenized in 50 mM sodium phosphate buffer (pH 7.8). After centrifugation at 12, 000*g* for 15 min at 4°C, the gained supernatant was used for the measurement of ascorbate, ROS and antioxidant enzyme activities.

The concentration of ascorbate (ASC) was measured using VC (vitamin C, ascorbic acid) Assay Kit (A009, Jiancheng, Nanjing, China). The principle of this assay is that Fe^3+^ reacts with the reduced form of ascorbic acid to become Fe^2+^, and the latter participates in the color reaction with phenanthroline. The content of ASC was calculated by the absorbance at 536 nm.

For H_2_O_2_ content analysis, 1 ml of the above supernatant was homogenized in 1 ml of 0.1% titanium sulfate mixed with 20% (v/v) H_2_SO_4_ thoroughly for 10 min. The absorbance of the gained supernatant was measured at 410 nm after centrifugation at 12, 000*g* for 10 min at room temperature (Hu et al., 2012). Then, the H_2_O_2_ level was calculated according to a standard curve of H_2_O_2._

In the study, the measured antioxidant enzymes included the total SOD, POD, and CAT. The total SOD and CAT activities were assayed by the Total SOD Assay Kit with WZT-1 (S0102, Beyotime, Shanghai, China) and CAT Assay Kit (S0051, Beyotime, Shanghai, China), respectively, on the basis of the manufacturer’s instructions. The POD activity was determined with Plant POD Assay Kit (A084-3, Jiancheng, Nanjing, China) according to the introduction.

### RNA Isolation and Real-Time Quantitative PCR

After drought stress treatment for 12 days, similar size plans from control and stress treatment conditions were selected for drought-related gene expression analysis. Total RNA from leaf, stem and root was extracted using Trizol reagent (Invitrogen, Carlsbad, CA, USA) and treated with RNase-free DNase (Promega, Madison, WI, USA). DNA-free total RNA was reverse-transcribed into first-strand cDNA with reverse transcriptase (TOYOBO, Ohtsu, Japan).

Quantitative real-time PCR was performed using CFX 96 Real Time System (Bio-Rad, Richmond, CA, USA) with SYBR-green fluorescence and the results were analyzed by comparative ΔΔCT method. Gene-specific primers for qRT-PCR were listed in **Table 1**. The thermal cycle used was 95°C for 5 min, 40 cycles of 95°C for 15 s, 55°C for 15 s, and 72°C for 30 s. All experiments were repeated three biological replicates and the relative transcript levels were standardized with *MsActin* as internal control.

**Table 1:** Gene-specific primers for qRT-PCR.

Gene	Primer sequence (5′to 3′)
*MtP5CS*	F: 5**′**-GAGAGGGAACGGCCAAGTG-3**′**
	R: 5**′**-CAGATCCTTGTGTGTATA-3**′**
*MtProDH*	F: 5**′**-CCAACGTCCACGCTGATAAGA-3**′**
	R: 5**′**-ACAGGTCCTATAGCCGTTGCA-3**′**
*MtCorA1*	F: 5**′**-GGCGGAGGTGGTTACAATGG-3**′**
	R: 5**′**-GGCAACAGATTCAGCAGCAC-3**′**
*MtDehyd*	F: 5**′**-GAGCGAGGAGGAAGTTGATGG-3**′**
	R: 5**′**-TGGTGCTGGTGGAGTTGTTA-3**′**
*MsNAC*	F: 5**′**-TGGCTTTAGATTTCATCCAACTG-3**′**
	R: 5**′**-AATACCATTCATTCTCCCCAAAC-3**′**
*MtCBF4*	F: 5**′**-GATTGCACTGAGAGGAAGGTC-3**′**
	R: 5**′**-CCGCCTTTTGAATATCCCTTG-3**′**
*MtRD2*	F: 5**′**-GCAGCTGTGGTTCTGGGGACC-3**′**
	R: 5**′**-AGCAATACTCACCGACGCTTCCT-3**′**
*MsHSP23*	F: 5**′**-CATTCAACACCAACGCCATG-3**′**
	R: 5**′**-CGGATCAAACACATCTGAGAGG-3**′**
*MsActin*	F: 5**′**-TCCTGGGTGCTCTTCAGGAGCAA-3**′**
	R: 5**′**-TAGGGCTGTGTTTCCAAGT-3**′**

### Statistical Analyses

All experiments in the study were repeated at least three times and the results explained are the mean ± SE of these independent experiments. The asterisk above the columns of figures indicated significant difference at *p* < 0.05 (Student’s *t*-test).

## Results

### Comparison of Water Status and Cell Membrane Damage of Two Alfalfa Varieties Under Drought Stress

**FIGURE 1:**
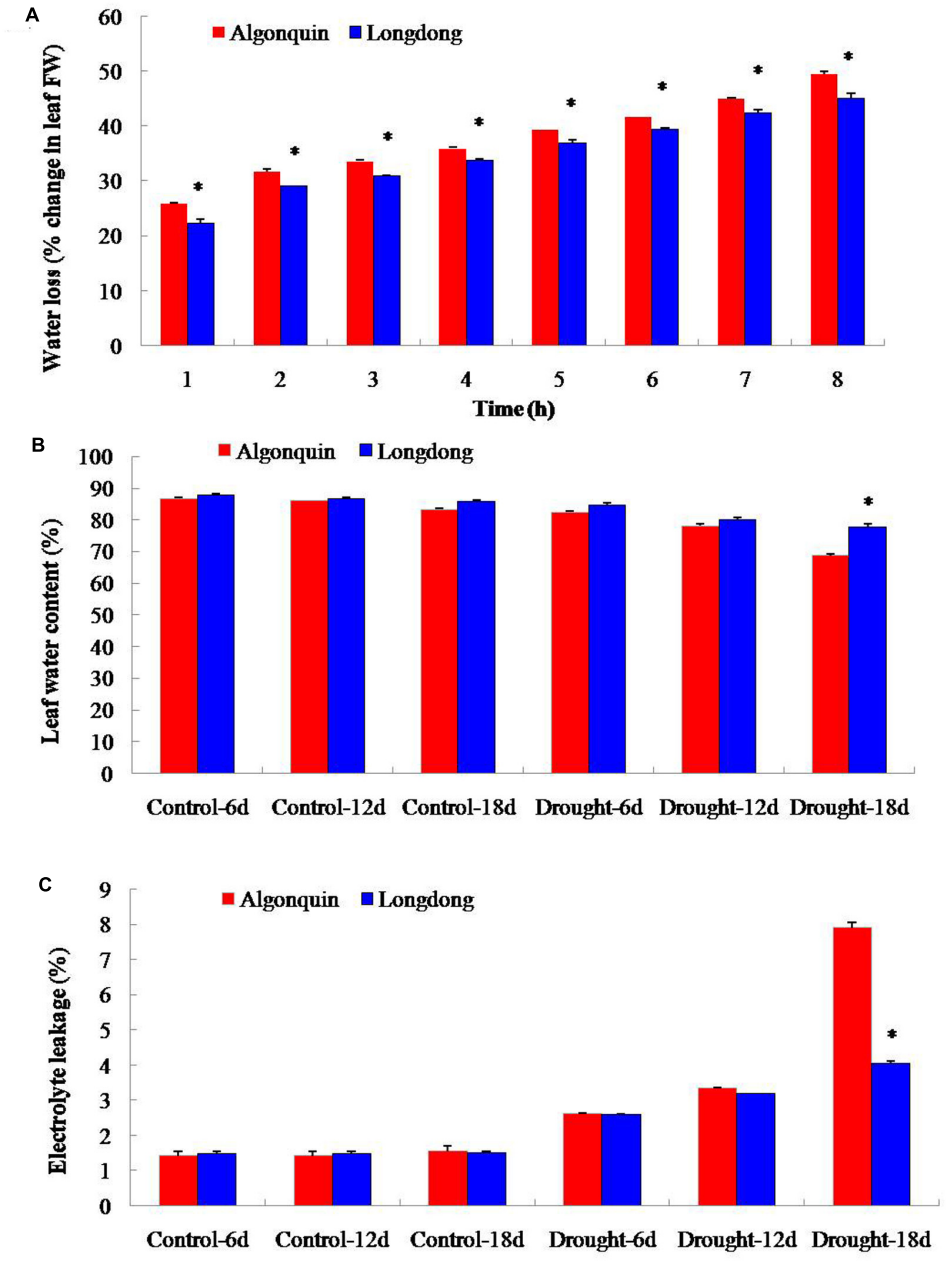
**Quantitative comparison of water status and electrolyte leakage (EL) of two alfalfa varieties differing in drought tolerance. (A)** At drought stress 12 days, water loss of Algonquin and Longdong was compared under control condition. **(B)** LWC under control and drought conditions. **(C)** EL under control and drought conditions. The results shown are means ± SE (*n* = 5). Asterisk symbols indicate significant differences from Algonquin at *P* < 0.05 (Student’s *t*-test).

To analyze the difference of drought tolerance between two alfalfa varieties, 4-week-old plants were, respectively, subjected to well-watered condition and drought condition by withholding water. Under control condition, the leaf water loss of Algonquin from 1 to 8 h after detachment ranged from 25.9 to 49.5%, which was significantly faster than that of Longdong ranged from 22.3 to 45.0% (**Figure 1A**). When subjected to drought stress, two varieties showed gradually decreased LWC; however, LWC of Longdong (78.0%) was significantly higher than that of Algonquin (68.8%) at 18 days after drought stress (**Figure 1B**). EL of two varieties under drought stress markedly increased, especially after drought stress for 18 days when compared to control condition. Additionally, Algonquin revealed significantly higher EL than Longdong at 18 days after drought stress (**Figure 1C**). These results suggested that Londong was more tolerant to drought than Algonquin partially due to slower water loss *in vitro*, higher water content *in vivo* and less cell membrane damage.

### Comparison of Stomatal Density Between Two Alfalfa Varieties

**FIGURE 2:**
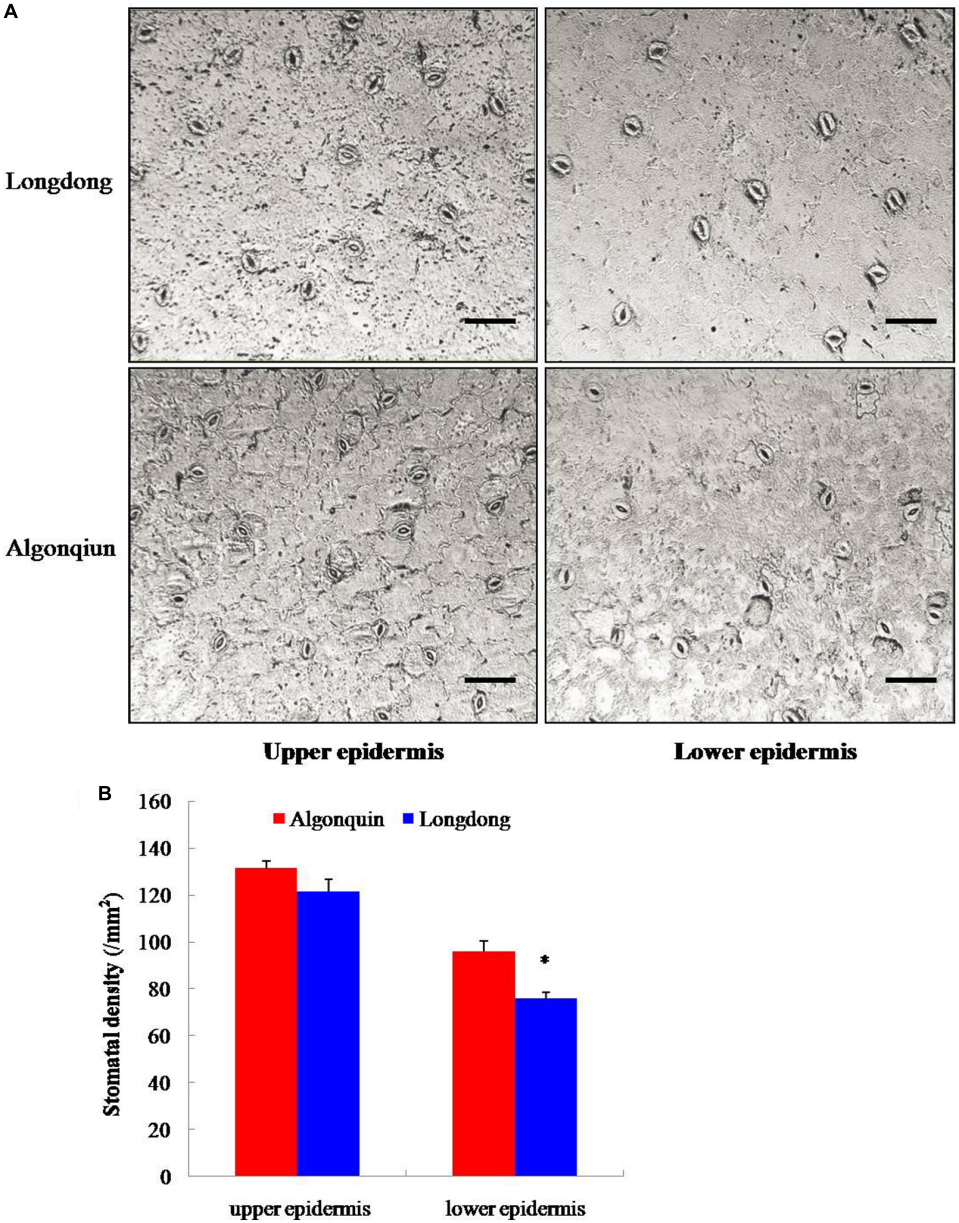
**Evaluation of stomatal density. (A)** The upper epidermis and lower epidermis of 4-week-old terminal leaflets were photographed by fluorescence microscope. Bars = 50 μm. **(B)** Stomatal density of well-watered Longdong and Algonquin (*n* = 15). The results shown are means ± SE. Asterisk symbols indicate significant differences at *P* < 0.05 (Student’s *t*-test).

To illustrate the physiological mechanism responsible for the contrasting drought tolerance of two alfalfa varieties, the stomatal density of well-watered alfalfa varieties at 4-week-old was studied (**Figure 2A**). The stomatal density of Longdong was 122 mm^-2^ and 76 mm^-2^ on the upper epidermis and lower epidermis, and that of Algonquin was 131 mm^-2^ and 96 mm^-2^, respectively (**Figure 2B**). Compared with Algonquin, Longdong displayed a lower stomatal density on both epidermises and significant difference was found on the lower epidermis. The results suggested that the lower stomatal density was one of the reasons for the lower water loss and higher LWC in Longdong compared with Algonquin (**Figures 1A,B**).

### Influence of Drought Stress on Leaf Size and Root Growth of Two Varieties

**FIGURE 3:**
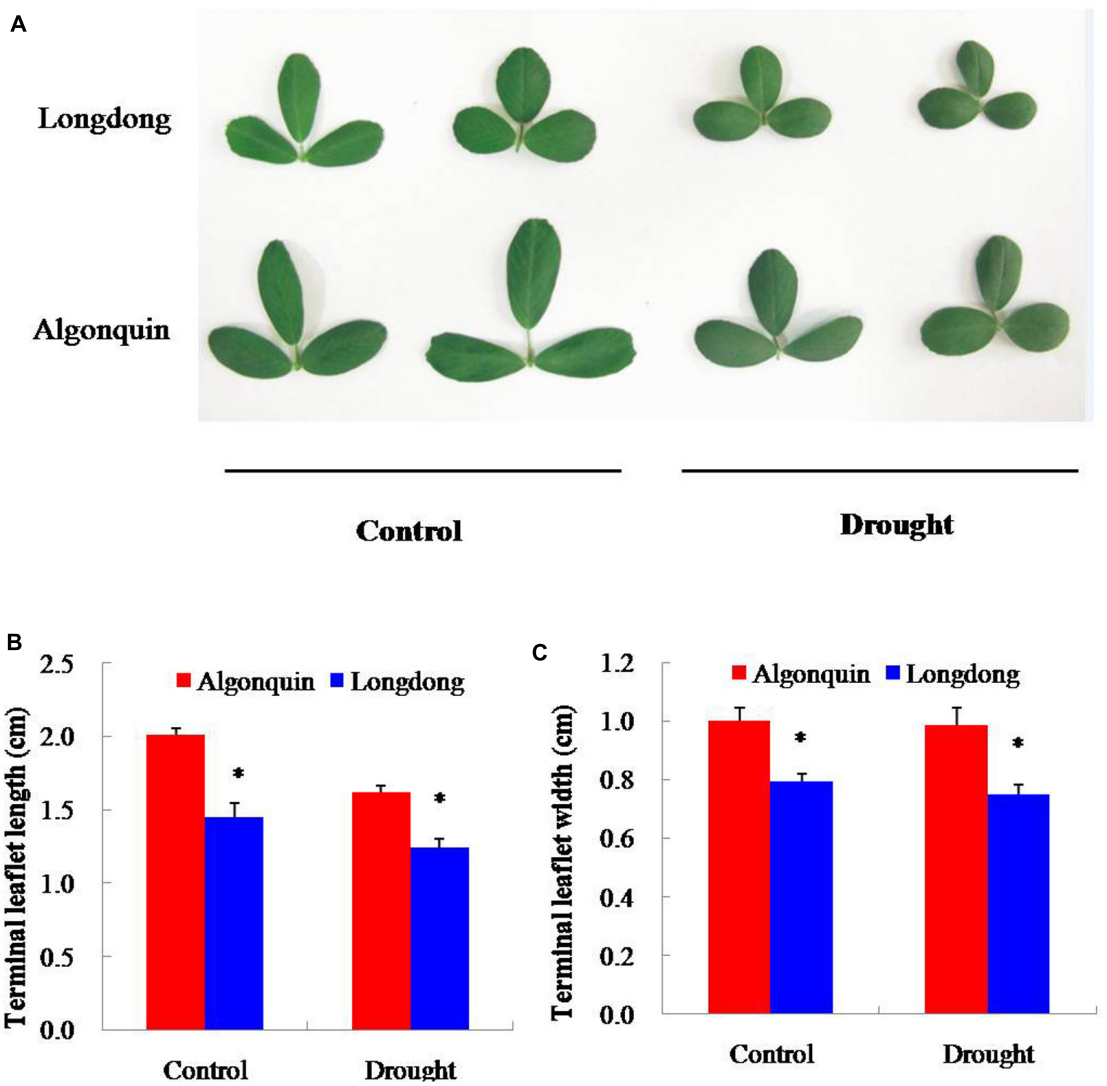
**Effect of drought stress on leaf size of two alfalfa varieties. (A)** The same part leaves of plant were collected from control and stress conditions after drought treatment 12 days. **(B,C)** Terminal leaflet length and width of two alfalfa varieties under control and drought conditions (*n* = 8). The results shown are means ± SE. Asterisk symbols indicate significant differences at *P* < 0.05 (Student’s *t*-test).

In the study, the effect of drought stress on terminal leaflet size was compared between the two varieties after 12 days of stress treatment (**Figure 3A**). Under drought condition, the terminal leaflet length of the both varieties was greatly decreased compared with control; however, the width of terminal leaflet showed a slight decrease. No matter under control or drought condition, significantly shorter length, and narrower width of terminal leaflet were found in Longdong compared to Algonquin (**Figures 3B,C**). The results indicated that Longdong had a smaller leaf size to reduce leaf area than Algonquin.

**FIGURE 4:**
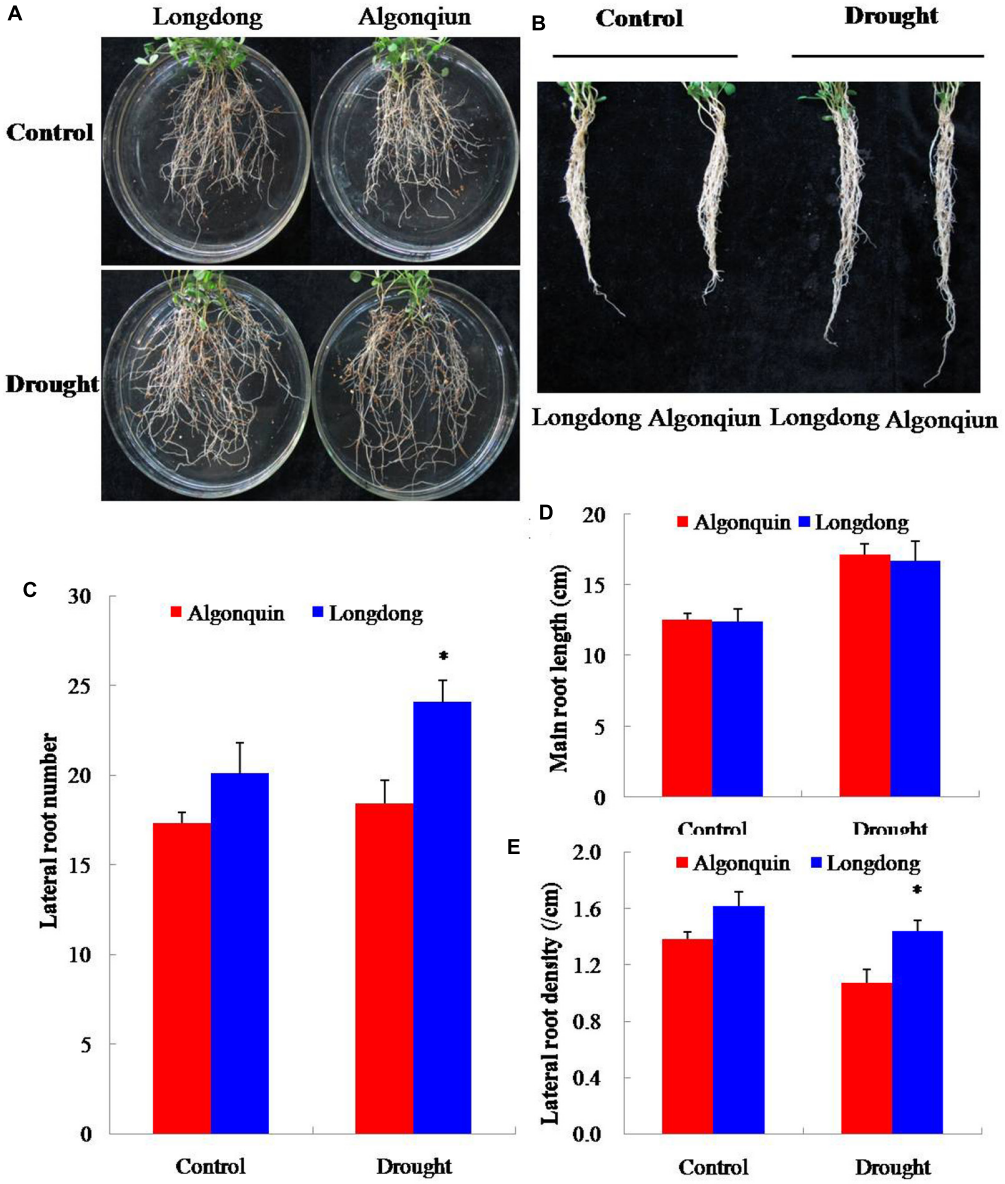
**Quantitative analysis of root growth in two alfalfa varieties. (A,B)** Roots of Longdong and Algonquin. The root systems of the two varieties were collected from control and stress conditions after drought treatment for 6 days. **(C–E)** Lateral root number, main root length and lateral root density between two varieties (*n* = 15). The results shown are means ± SE. Asterisk symbols indicate significant differences from Algonquin at *P* < 0.05 (Student’s *t*-test).

To examine the impact of drought stress on root growth, 2-week-old alfalfa seedlings were subjected to drought stress. After 6 days of growth, the lateral root numbers were counted and the main root length was measured. Treatment with drought resulted in an obvious increase in the lateral root numbers and the main root length of the two varieties (**Figure 4**). More lateral root numbers were observed in Longdong under control and drought conditions than Algonquin, and significant difference was found after drought stress (**Figure 4C**). No obvious difference was found in the main root length between the two varieties under control and drought conditions (**Figure 4D**). Accordingly, Longdong showed greatly higher lateral root density than Algonquin, especially under drought condition (**Figure 4E**). Therefore, Longdong with more lateral root number exhibited increased superficial area of root system which might contribute to more water absorbance from the soil during water deficiency compared with Algonquin.

### Effect of Drought Stress on Plant Growth of Two Alfalfa Varieties

**FIGURE 5:**
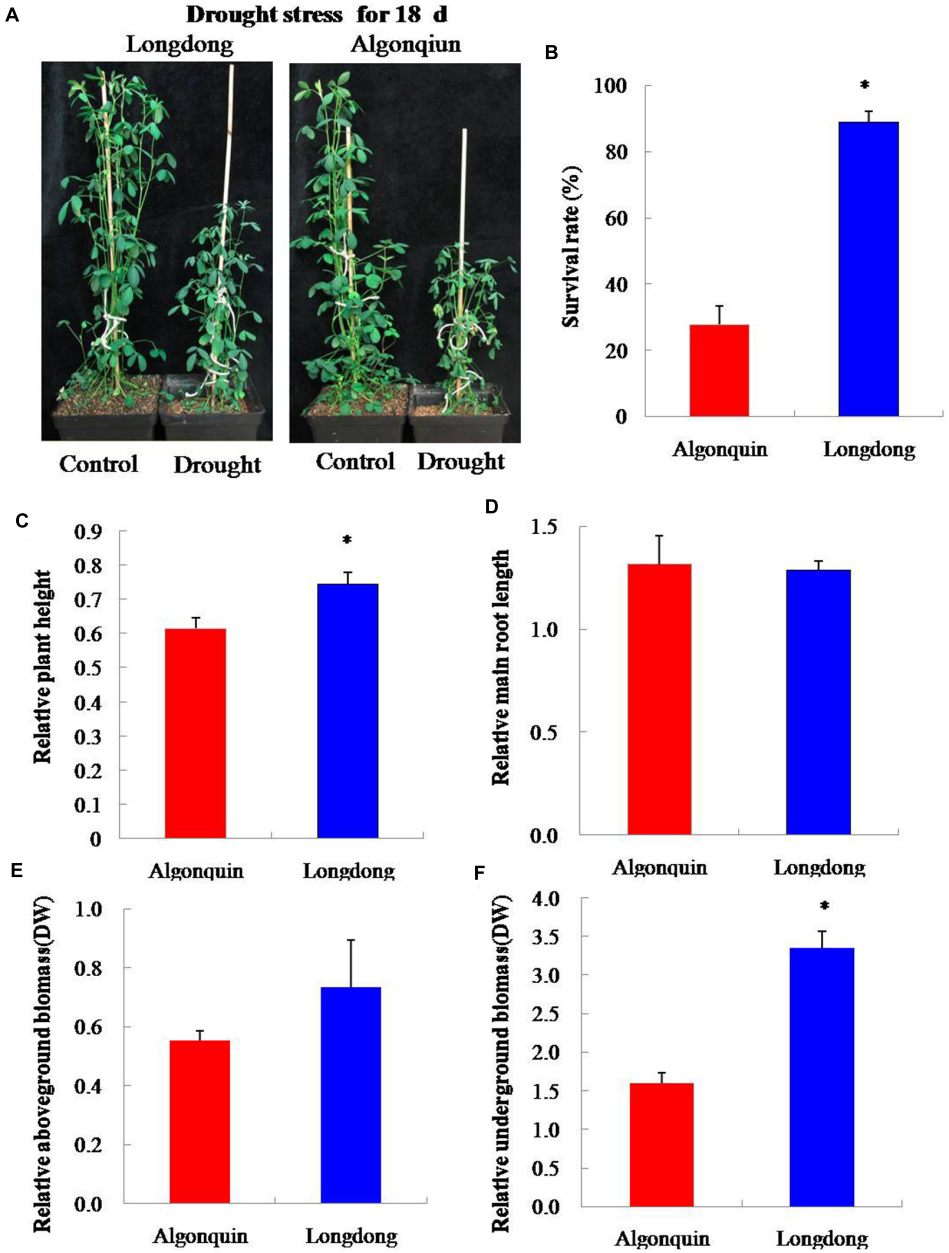
**Plants response to drought stress. (A)** Four-week-old plants of Algonquin and Longdong treated with drought stress for 18 days. Plants were photographed after 18 days stress treatment. **(B)** Survival rates of two varieties after re-watering 7 days were calculated from the results of three independent experiments (*n* = 20). **(C,D)** Relative plant height and relative main root length were compared after drought treatment 18 days relative to control (*n* = 8). **(E,F)** After drought treatment 18 days, the relative aboveground and underground biomass (DW, dry weight) per plant were analyzed under drought stress relative to control (*n* = 8). The results shown are means ± SE. Asterisk symbols indicate significant differences from Algonquin at *P* < 0.05 (Student’s *t*-test).

After drought stress for 18 days, we investigated the impact of drought stress on plant growth of two contrasting varieties. Algonquin showed more severe phenomena of leaf firing than Longdong (**Figure 5A**). The survival rate of Longdong was 89.0%, while that of Algonquin was only 27.8% after re-watering for 7 days (**Figure 5B**). Moreover, the relative plant height of Longdong was significantly higher than that of Algonquin after 18 days of drought stress relative to control (**Figure 5C**). Additionally, no significant difference was observed for the relative main root length under drought condition relative to control between two varieties (**Figure 5D**). Less leaf firing and relative higher plant height resulted in much more relative aboveground biomass per plant of Longdong than that of Algonquin under stress condition (**Figure 5E**). For the relative underground biomass per plant, Longdong showed significantly more dry weight by drastically increased lateral root numbers under drought condition than Algonquin (**Figures 4C** and **5F**). Taken together, Longdong suffered less harmful effect by drought stress on plant growth and had higher relative biomass during water deficiency compared with Algonquin.

### Changes of Proline and ASC Contents of Two Alfalfa Varieties Under Drought Stress

**FIGURE 6:**
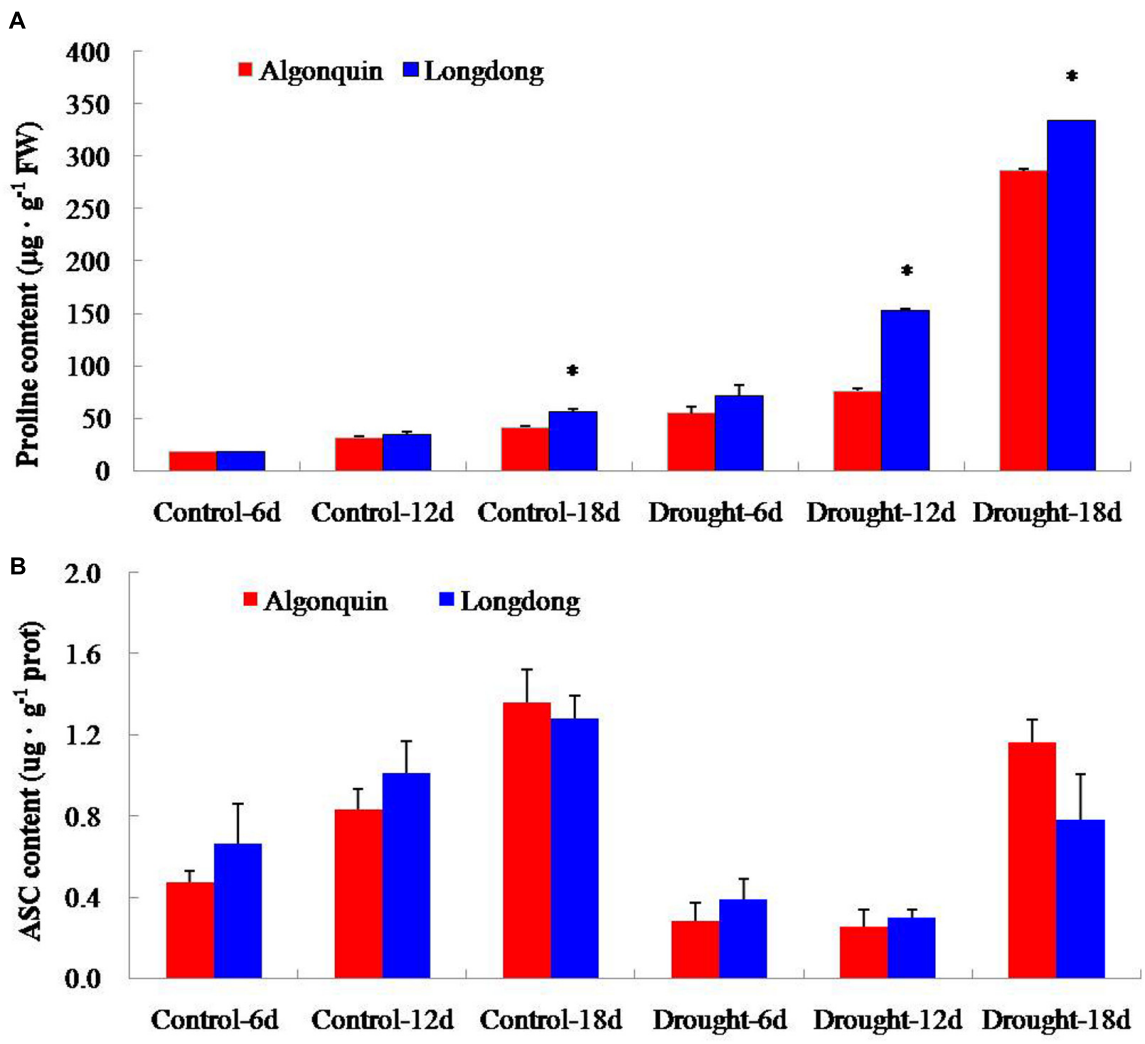
**The contents of proline and ASC of two varieties affected by drought. (A,B)** The accumulation of proline and ASC of two alfalfa varieties during drought stress. The results shown are means ± SE (*n* = 3). Asterisk symbols indicate significant differences from Algonquin at *P* < 0.05 (Student’s *t*-test).

Under well-watered condition, the internal proline content of two varieties was very low; and interestingly, the proline content in Longdong was higher than Algonquin. After drought stress treatment, proline content showed an obvious increase in both varieties; moreover, Longdong exhibited significantly higher proline content than Algonquin at drought treatment 12 and 18 days (**Figure 6A**). With the plant growth, the ASC content in both varieties showed an obvious increase under control condition; and Longdong revealed more ASC content than Algonquin expect that at 18 days. The content of ASC displayed a great decrease when subjected to drought stress in two varieties and more ASC content was also found in Longdong expect that at 18 days of drought stress (**Figure 6B**). Together, the higher proline and ASC contents in Longdong might partially react to better retain water in cells and protect cells from oxidative damage associated with drought.

### Changes of H_2_O_2_ Accumulation of two Alfalfa Varieties Under Drought Stress

**FIGURE 7:**
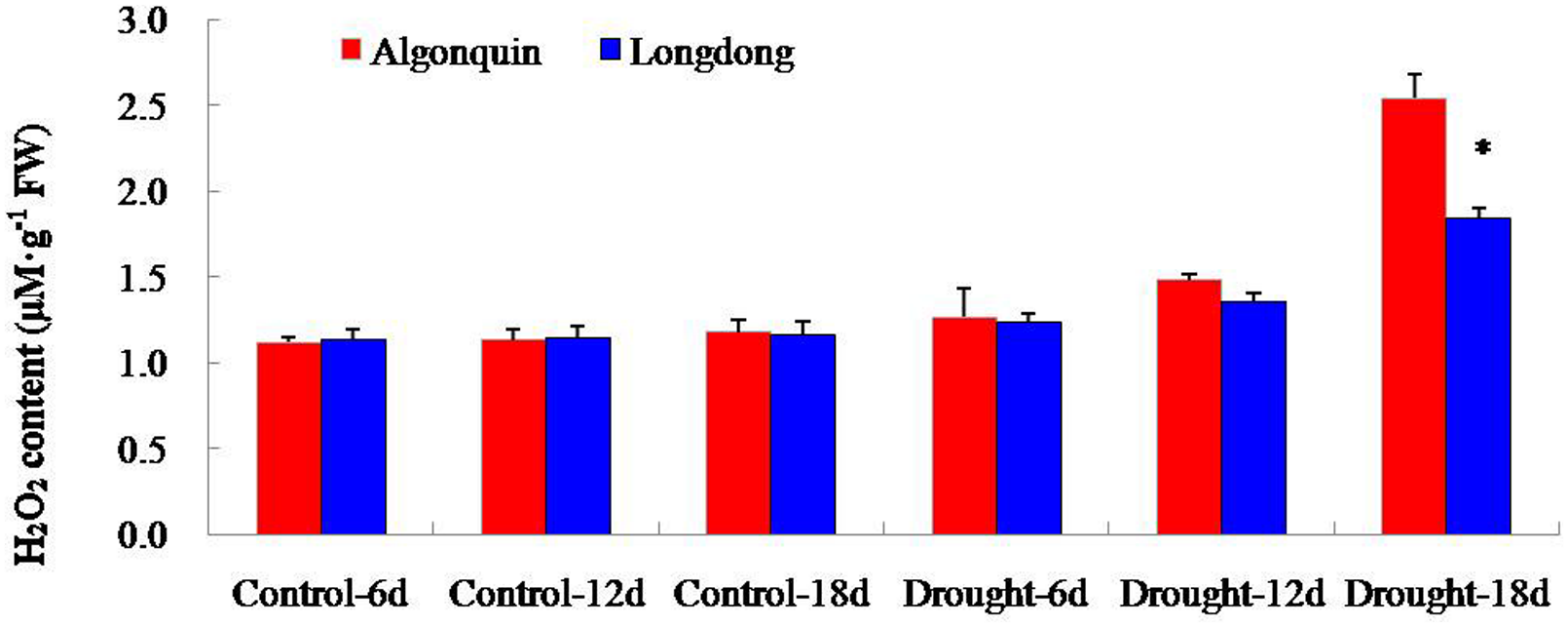
**Changes of H_2_O_2_ level in two alfalfa varieties after drought treatment**. The results shown are means ± SE (*n* = 3). Asterisk symbols indicate significant differences from Algonquin at *P* < 0.05 (Student’s *t*-test).

To investigate the differential oxidative damage suffered from drought stress in two alfalfa cultivars, we analyzed the content changes of H_2_O_2_ (**Figure 7**). H_2_O_2_ content of Algonquin and Longdong was similar under control and after drought stress for 6 and 12 days, but showed a large increase at drought stress 18 days. Longdong exhibited significantly lower H_2_O_2_ content than Algonquin (**Figure 7**).

### Changes of Antioxidant Enzyme Activities of Two Alfalfa Varieties Under Drought Stress

**FIGURE 8:**
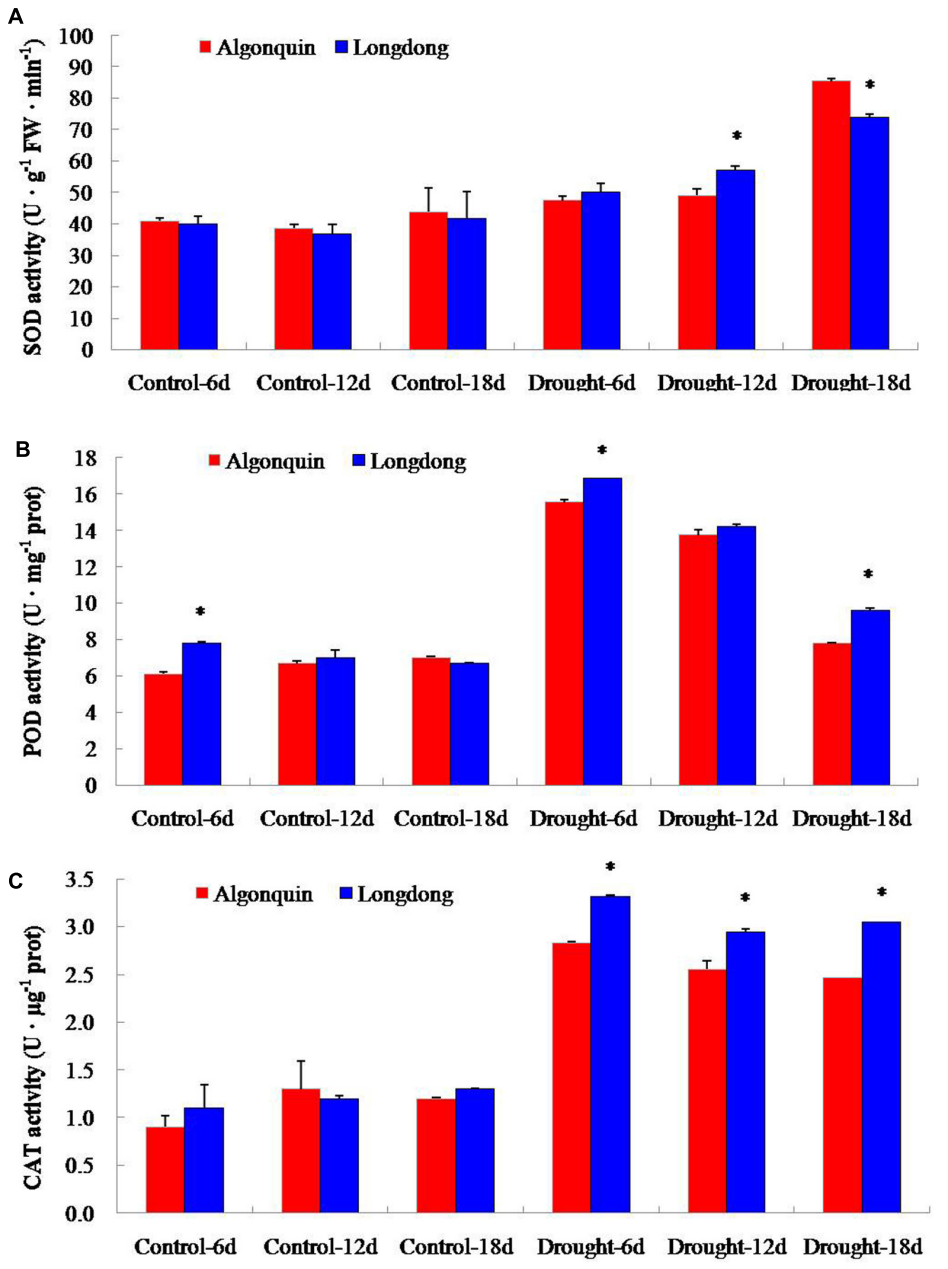
**Effect of drought stress on antioxidant enzyme activities. (A–C)** SOD, POD, and CAT activities of two varieties during drought stress. The results shown are means ± SE (*n* = 3). Asterisk symbols indicate significant differences from Algonquin at *P* < 0.05 (Student’s *t*-test).

In order to characterize the changes of antioxidant enzyme activity of the two alfalfa varieties to long- term drought stress, we measured the activity alterations of total SOD, POD and CAT. Under control condition, the activity of SOD was similar in both cultivars. Obviously, drought stress treatment enhanced the activity of SOD in two varieties, and Longdong showed significantly higher SOD activity than Algonquin at drought stress 6 and 12 days (**Figure 8A**). The activity of POD in two varieties sharply increased at drought stress 6 days; and then it showed a gradually decline at drought stress 12 and 18 days. Furthermore, Longdong exhibited significantly higher POD activity compared to Algonquin at drought stress 6 and 18 days (**Figure 8B**). Similar to the change of POD activity, drought stress resulted in an obvious increase of CAT activity in both varieties. Significant higher activity of CAT was found in Longdong than in Algonquin during the whole drought process (**Figure 8C**).

### Comparative Expression Analysis of Stress-Responsive Genes

The expression of numerous plant genes has been reported to be regulated by drought stress (Zhu et al., 1997), including the large number of osmolyte biosynthesis genes, LEA/dehydrin-type genes, detoxification enzymes, chaperones, proteases, and ubiquitination-related enzymes (Zhu, 2002). In the present study, we analyzed the expression changes of eight drought-responsive genes in leaf and root by real-time RT-PCR at 12 days after drought stress.

**FIGURE 9:**
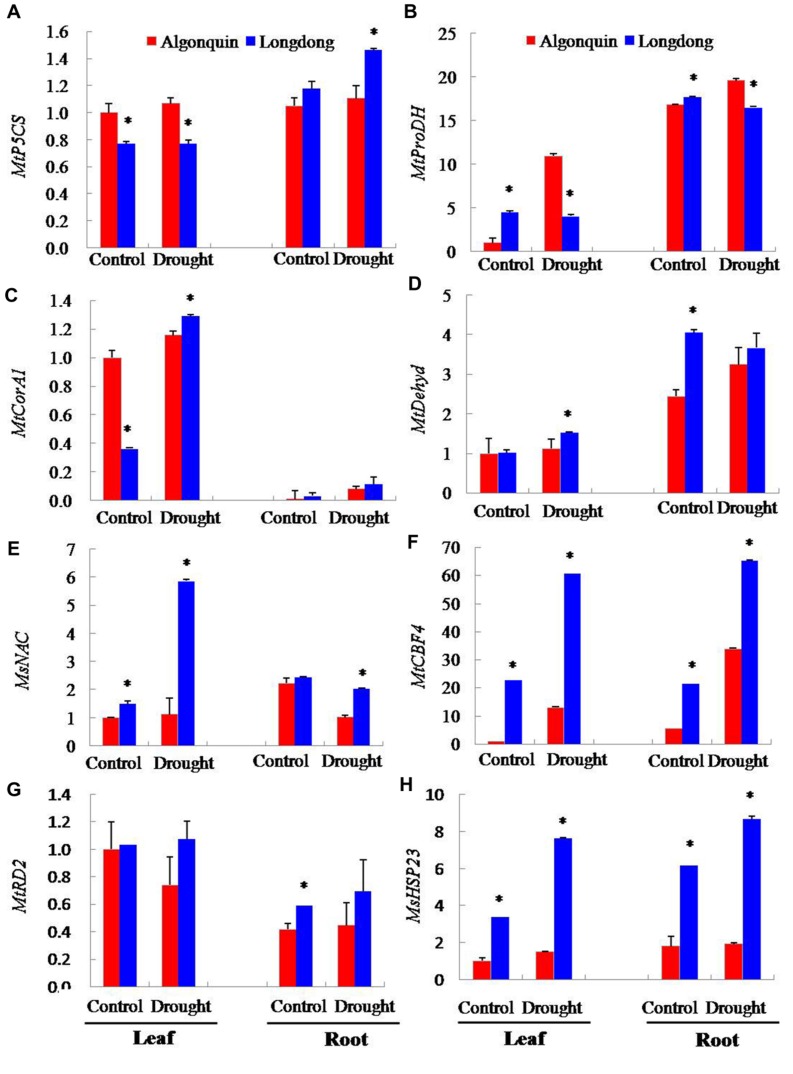
**Transcriptional expression of drought responsive genes in leaf and root after drought stress. (A–H)** Changes in expression of *MtP5CS*, *MtProDH*, *MtCorA1*, *MtDehyd*, *MsNAC*, *MtCBF4*, *MtRD2*, and *MsHSP23* genes in leaf and root of alfalfa plant after 12 days of drought treatment. The results shown are means ± SE (*n* = 3). Asterisk symbols indicate significant differences at *P* < 0.05 (Student’s *t*-test).

Under control condition, the transcripts of *MtP5CS*, *MtProDH*, *MtDehyd*, *MsNAC*, and *MsHSP23* in both varieties were significantly higher in root than in leaf, while *MtCorA1* and *MtRD2* showed significantly higher expression in leaf than in root (**Figure 9**). After drought stress, the expression of eight genes was changed and induced by water deficiency (**Figure 9**). In response to drought stress, the expression of *MtP5CS* in root of Longdong was significantly increased and was higher than that of Algonquin; however, in leaf, no obvious changes were found between two varieties compared to control (**Figure 9A**). In addition, Longdong showed significantly lower expression of *MtProDH* than Algonquin in both leaf and root under drought condition, although the gene expression in Longdong was obviously higher than that in Algonquin under control condition no matter in leaf or root (**Figure 9B**). Drought stress induced higher expression of *MtCorA1*, *MtDehyd*, and *MtRD2* in both Longdong and Algonquin. Moreover, the expression levels of the three genes in Longdong were obviously higher than that in Algonquin in both leaf and root under drought condition (**Figures 9C,D,G**). The expression levels of *MsNAC*, *MtCBF4*, and *MsHSP23* genes were also largely induced by drought stress in two varieties, and significantly higher expression levels of these genes were found in Longdong than in Algonquin no matter under control or drought condition (**Figures 9E,F,H**).

## Discussion

The responses of plants to drought stress have been observed at many levels, from physiological and biochemical to genetic and developmental level, and from the cellular to the whole-plant level for acclimatization, survival and reproduction, ultimately (Kang et al., 2011). These responses include, for example, leaf wilting, reduction in leaf area, stimulation of root growth, changes in relative water content and membrane structure, generation of reactive oxygen species, and accumulation of osmolytes and antioxidants, and transcriptional activation of drought-responsive genes (Miller et al., 2010; Kang et al., 2011; Lata and Prasad, 2011). Selection of naturally stress tolerant genotypes among different varieties is the lowest cost and the most efficient method for plant breeding (Luo et al., 2011). Alfalfa, with significant economic value and excellent agricultural traits, is one of the most important legume forage crops, and has natural variation in the drought response (Chao et al., 2009; Kang et al., 2011). In this study, we performed an integrated and comparative analysis of two alfalfa varieties with different drought tolerance on the basis of physiological, morphological, and molecular changes.

Plant acclimates to drought stress effectively by reducing transpirational water loss, which maintains soil moisture and keeps plants to conserve an adequate water status to sustain critical physiological and biochemical processes (Yoo et al., 2010). The leaf water loss *in vitro* and the LWC *in vivo* reflect the water status of plant and water loss is a key for plant survival during drought stress condition (Hu et al., 2012; Shi et al., 2012a). Stomatal conductance regulates transpirational flux and water use, and is dependent on stomatal density and movement (Kim et al., 2010). Previously, a lower stomatal density leads to the reduced water loss and confers higher drought resistance has been demonstrated (Ouyang et al., 2010). *MtCAS31* overexpression dramatically diminished stomatal density and markedly promoted the drought tolerance of transgenic *Arabidopsis* (Xie et al., 2012). The lower stomatal density in Longdong variety under well-watered condition may account for the significantly lower water loss and higher LWC than Algonquin under drought condition (**Figures 1A,B** and **2**). Meanwhile, Longdong showed smaller leaf size compared with Algonquin, contributing to reduce the area of transpiration under drought stress (**Figure 3**). The results indicated that Longdong could maintain higher water status to alleviate water deficiency and had higher resistance to drought compared with Algonquin.

When subjected to drought stress, plants decreased the aboveground biomass, in part, as a consequence of an investment in root growth, which leaded to an increase in underground biomass under drought condition relative to control (**Figures 5E,F**). Developing a larger root system enhances water uptake under field conditions, and becomes a common strategy in plants for drought avoidance (Yamaguchi and Sharp, 2010). Although the main root length of Algonquin is similar to Longdong, more lateral root numbers and higher density of lateral root were found in Longdong, which was conducive to absorb more water from the soil under water deficiency than Algonquin (**Figure 4**).

Drought stress has direct effect on the disturbance of cell membrane and EL has been widely used to evaluate the extent of cell damage under various hostile environments (Zhao et al., 2011). Drought tolerant bermudagrass variety exhibited relative lower water loss, higher LWC and less severe cell membrane than drought sensitive variety under drought stress condition (Shi et al., 2012a). In the study, lower EL was observed in drought-tolerant Longdong variety than drought-sensitive Algonquin variety, suggesting that Longdong suffered less cell injury from drought stress (**Figure 1C**). These above results were consisted with the higher survival rate of Longdong with less leaf firing after drought stress (**Figures 5A,B**).

Plants synthesize a variety of osmolytes that lower water potential and retain water uptake under stress environment. Previous reports on osmolyte accumulation in alfalfa primarily focused on proline, which has been reported to accumulate in the leaf, root, nodules, and phloem sap of drought-stressed alfalfa plants (Aranjuelo et al., 2011; Kang et al., 2011). Increased proline accumulation provides a significant superiority for plants to avoid different stresses by increasing the osmotic turgor inside plant cells and absorbing more water to keep a significantly increase in LWC (Zhao et al., 2011; Shi et al., 2012a). After drought stress, drought tolerant plant variety showed significantly higher proline content than drought sensitive variety (Lu et al., 2009; Shi et al., 2012a). Consistently, drought tolerant Longdong accumulated significantly higher proline content than drought sensitive Algonquin, indicating that this variety maintained higher cell membrane integrity, partially accounting for lower EL and higher LWC under drought condition (**Figures 1** and **6A**).The adverse environments always cause the over-production of ROS such as H_2_O_2_, HO^•^ and O_2_^−^, leading to lipid peroxidation, protein oxidation, DNA fragmentation, enzyme inhibition, and cell death, ultimately (Apel and Hirt, 2004; Wang et al., 2009). Previous researches revealed that stress-tolerant cultivars showed a higher tolerance to oxidative damage under stress (Naya et al., 2007; Wang et al., 2009). A higher accumulation of ROS might account for a greater ROS-induced damage in the drought-sensitive cultivar compared to the tolerant cultivars (Pyngrope et al., 2013). Significant lower H_2_O_2_ level was observed in tolerant Longdong after 18 days of treatment, suggesting that the variety suffered less oxidative damage from drought stress (**Figure 7**), which might be responsible for the lower EL of Longdong compared to Algonquin (**Figure 1C**). To scavenge the excessive ROS and to avoid these deleterious effects, plants possess a complex antioxidative defense systems comprising of the non-enzymatic and enzymatic antioxidants, which are critical for the survival under stress conditions (Mittler, 2002; Foyer and Noctor, 2005; Sharma et al., 2012). The reduced form of vitamin C, ASC, is a powerful antioxidant because it can directly interact with and detoxify ROS, and thus contribute significantly to non-enzymatic ROS scavenging (Frikke-Schmidt et al., 2016). With imposition of water deficit, the level of ASC showed a decline, especially significantly in drought-sensitive variety; the drought tolerant variety possess the higher capacity to maintain higher level of ASC in order to scavenge ROS and resist the harmful condition (Pyngrope et al., 2013). Higher ASC contents in transgenic plants exhibited enhanced tolerance to oxidative stress (Wang et al., 2010). Higher levels of ASC in drought-tolerant Longdong at drought stress 6 and 12 days contributed to scavenge ROS resulted from water deficiency when compared to the sensitive Algonquin (**Figure 6B**). In addition, SOD is the key enzyme in the active oxygen-scavenging system and catalyzes the dismutation reaction of O_2_^−^ into H_2_O_2_ and O_2_. POD and CAT are responsible for detoxification of H_2_O_2_, leading to reduce H_2_O_2_ levels. Increased activities of the enzymes would decrease ROS levels (Apel and Hirt, 2004; Ouyang et al., 2010). Higher antioxidant enzyme activities were found in tolerant species than in sensitive species under various environmental stresses (Jagtap and Bhargava, 1995; Türkan et al., 2005; Wang et al., 2009). Consistently, we found higher total SOD, POD, and CAT activities in leaf of Longdong than that of ofAlgonquin under drought stress condition (**Figure 8**). In accordance with lower ROS levels, these results indicated that the drought-tolerant Longdong possessed a better ROS scavenging ability compared with drought-sensitive Algonquin.

Abiotic stresses regulate the expression of thousands of genes in plants at both the transcriptional and the posttranscriptional levels (Long et al., 2013). In the present study, we detected the transcriptional expression variation of eight stress -related genes under drought condition (**Figure 9**). Stress conditions could induce proline accumulation in plants. It has been showed that proline acts as an osmoprotectant and antioxidant to scavenge free radicals and also functions as a molecular chaperone to protect protein integrity and enhance the activities of different enzymes, thus conferring plant tolerance to environmental stresses (Ashraf and Foolad, 2007; Szabados and Savouré, 2010). The *MtP5CS* gene, which has been isolated from the model legume *M. truncatula*, plays a predominant role in stress-induced proline accumulation (Kam and Nam, 2013). Meanwhile, proline degradation is mediated by the action of proline dehydrogenase (ProDH; Szabados and Savouré, 2010). Greatly higher expression level of *MtP5CS*, especially in root, and obviously lower transcript of *MtProDH* in both leaf and root of Longdong (**Figures 9A,B**) might explain greater proline accumulation compared to Algonquin after drought stress (**Figure 6A**).

*MtCorA1* and *MtDehyd* encode a cold-inducible CORA protein and a dehydrin-related protein, respectively. Significantly higher expression of the *MtCorA1* and *MtDehyd* genes was observed in the salt-tolerant Jemalong A17 variety compared to salt-sensitive 108-R variety under salt stress (De Lorenzo et al., 2007). In the present study, we also found that the transcriptional expression of the two genes in drought-tolerant Longdong was significantly higher than in drought-sensitive Algonquin in both leaf and root under drought stress (**Figures 9C,D**). The C-repeat binding factor/dehydration responsive element binding factor (*CBF/DREB*), *MYB* and *CUC* (*NAC*) transcription factors have been described as important regulators in plant responses to environmental stress (Lata and Prasad, 2011). Transgenic *Arabidopsis* with *MsNAC* and *MtCBF4* genes, respectively, has the better drought tolerance with activating expression of downstream genes than the wild-type (Li et al., 2011; Wang, 2013). Drought stress significantly increased the expression level of *MsNAC* in leaf of Logndong, and the *MtCBF4* expression was obviously induced by drought stress in both varieties and was significantly higher in Longdong than in Algonquin (**Figures 9E,F**). Additionally, *MtRD2*, acting as a stress-responsive marker gene, was induced and the fold-inductions in transgenic alfalfa was greater than that of WT at some time points during drought process (Tang et al., 2013). Consisted with previous results, drought stress lead to higher increased expression of *MtRD2* in both leaf and root of Longdong (**Figure 9G**). Heat shock proteins (HSPs) are critical for protecting cells against damage caused by environmental stresses in a wide range of organisms. Overexpression of alfalfa mitochondrial *HSP23* confers enhanced tolerance to abiotic stress in transgenic tobacco and tall fescue (Lee et al., 2012a,b). The high levels of *MsHSP23* proteins in the transgenic plants protect cells from oxidative damage through chaperon and antioxidant activities (Lee et al., 2012b). In this study, drought stress resulted in increased expression of *MtHSP23*, and Longdong showed significantly higher *MtHSP23* expression in both leaf and root compared to Algonquin under drought stress (**Figure 9H**). Taken together, significantly higher expression of the drought-related genes was found in Longdong than in Algonquin, indicating that this variety may be more positively response to stress signal by accumulating more osmoprotectants and activating higher expression of downstream genes, which in turn may explain the better growth of Longdong under drought stress.

## Conclusion

**FIGURE 10:**
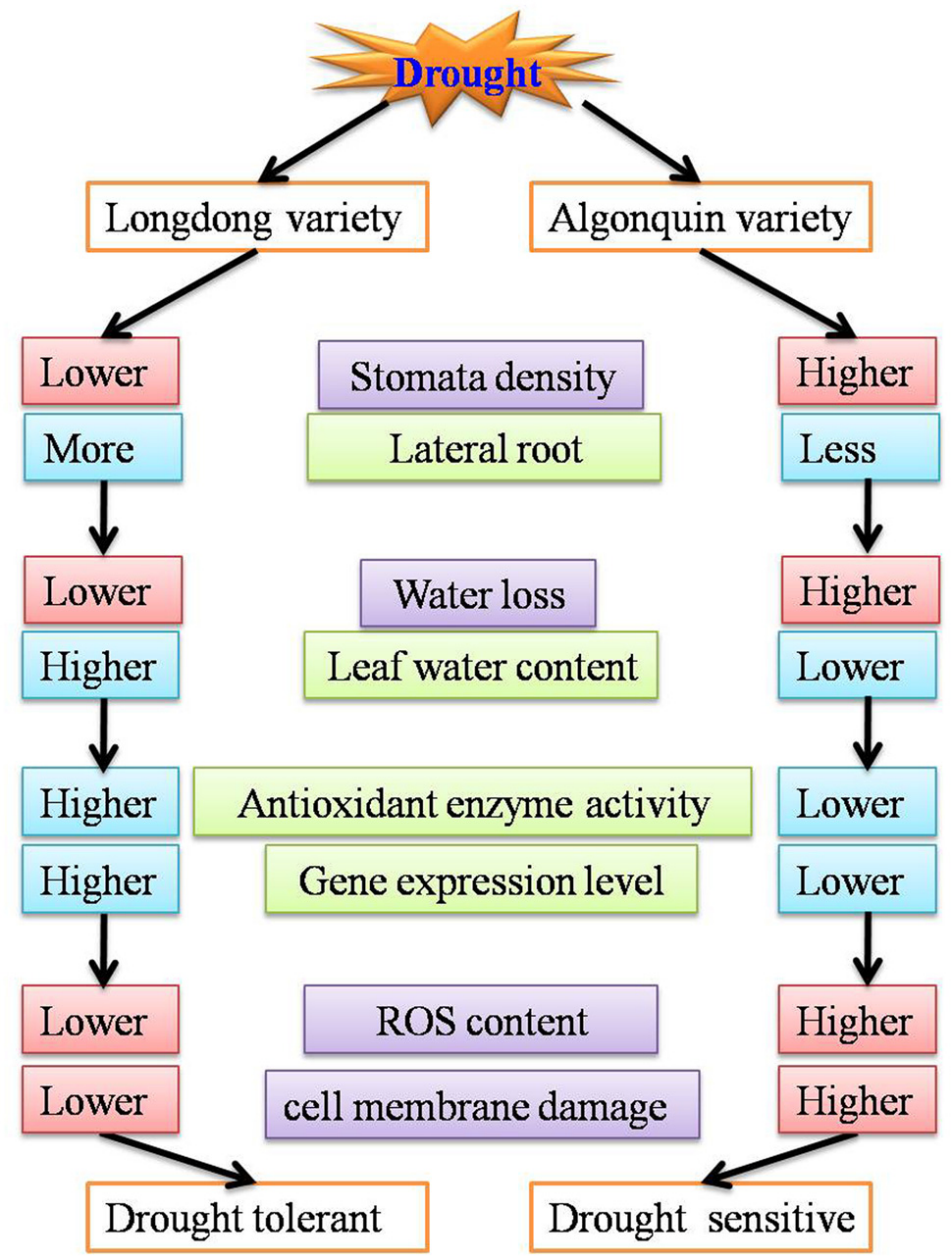
**A model depicting drought tolerance of two contrasting alfalfa varieties**.

In the study, our results showed that Longdong variety was more tolerant to drought stress than Algonquin variety as evidenced by quantitative differences at physiological, morphological and transcriptional levels. Longdong variety exhibited lower stomata density and more lateral roots which resulted in less water loss and higher LWC. In addition, compared to Algonquin, higher proline content and higher antioxidants may contribute Longdong to protect membrane stability and photosynthetic machinery from oxidative damage associated with drought stress, which might be responsible for the lower EL and less accumulation of H_2_O_2_ in Longdong. Meanwhile, stress-tolerant Longdong showed significantly higher expression of drought responsive genes, indicating that Longdong variety exhibits better genetic basis against drought stress than Algonquin variety (**Figure 10**). These results were expected to be valuable to breeding programs of novel alfalfa varieties with enhanced drought tolerance and improved matter production under adverse environments in future.

## Author Contributions

WQ and ZC designed the experiment; WQ and XL performed the experiments and analyzed the data; HW provided plant materials; HW and ZC guided the research; WQ and ZC wrote the manuscript.

## Conflict of Interest Statement

The authors declare that the research was conducted in the absence of any commercial or financial relationships that could be construed as a potential conflict of interest.
